# Internet Usage Habits and Experienced Levels of Psychopathology: A Pilot Study on Association with Spontaneous Eye Blinking Rate

**DOI:** 10.3390/jpm11040288

**Published:** 2021-04-09

**Authors:** Dovile Simkute, Igor Nagula, Povilas Tarailis, Julius Burkauskas, Inga Griskova-Bulanova

**Affiliations:** 1 Institute of Biosciences, Life Sciences Centre, Vilnius University, Sauletekio Av. 7, LT-10257 Vilnius, Lithuania; dovile.simkute@gmc.vu.lt (D.S.); igor.nagula@stud.gmc.vu.lt (I.N.); povilas.tarailis@stud.gmc.vu.lt (P.T.); 2 Laboratory of Behavioral Medicine, Neuroscience Institute, Lithuanian University of Health Sciences, Vyduno Str. 4, LT-00135 Palanga, Lithuania; julius.burkauskas@lsmuni.lt

**Keywords:** spontaneous eye blinking rate, sEBR, problematic internet use, PIU, PIUQ-9

## Abstract

Increasing availability of the internet has resulted in the increased prevalence of problematic online behaviors. Reliable and affordable neurobiological and psychological biomarkers that distinguish problematic internet use (PIU) from functional online activities are of utmost importance. Previous studies have shown a relationship between spontaneous eye blinking rate (sEBR) and changes in dopamine regulation in neurological and psychiatric disorders, including substance use disorders. In this study, we utilized sEBR to examine the potential link between individual differences in dopaminergic neurotransmission and PIU. In sum, 62 subjects participated in this study (median age 25, IQR 6 years, 34 females). The Problematic Internet Use Questionnaire (PIUQ-9), Beck Depression Inventory (BDI-II), Beck Anxiety Inventory (BAI), Clark–Beck Obsessive–Compulsive Inventory (CBOCI) and Barratt Impulsiveness Scale (BIS-11) were used for psychological assessment. The sEBRs were assessed with an electrooculogram recorded from above and below the left eye and from the right and left outer canthi. The group with PIU (PIUQ-9 > 20) expressed higher levels of impulsivity and compulsive behavior symptoms than the control group. In the group with PIU, impulsivity levels were inversely related to sEBR, and a trend of negative association of sEBR with compulsive behavior was observed. Future research should enroll subjects with high levels of PIU and strongly expressed psychopathology levels to further address the utility of sEBR as a potential biomarker.

## 1. Introduction

The increasing availability of the internet has resulted in the increased prevalence of problematic internet use (PIU) observed in young adults [1]. Extensive internet use has previously been associated with higher prevalence of depressive, anxiety symptoms [2,3], higher impulsivity rates [4], and certain personality traits [5]. Nevertheless, there is no consensus on diagnostic criteria and related terminology, which makes early risk detection and intervention difficult and complicates synthesis of the research findings related to PIU [6,7,8]. The most up-to-date strategy defines internet-related behaviors as a continuum, ranging from healthy to problematic or excessive use [9]. However, as the amount of time spent online by itself does not necessarily indicate problematic behavior [10], reliable and affordable neurobiological and psychological biomarkers that distinguish PIU from healthy/functional use are of utmost importance.

Accumulating evidence points at similarities between substance use disorders and behavioral addictions; these are observed on both behavioral and neural levels. Subjects with addictions frequently display a dysregulation of dopaminergic transmission. A reduced dopamine receptor availability in the striatum was shown in substance use disorders [11,12,13] and in PIU [14,15], suggesting that dopamine-related measures can be sensitive to the extent of behavioral problems. However, in humans, evaluation of dopaminergic transmission is only possible with expensive techniques such as positron emission tomography (PET), which also requires complex infrastructure and in-depth methodological expertise. Yet, spontaneous eye blinking rate (sEBR) was suggested as a potential, non-invasive, indirect marker of central dopamine function almost 40 years ago [16] with higher EBR indicating a higher level of dopaminergic signal transmission.

A review by Jongkees and Colzato [17] provided evidence in support of sEBR as a useful predictor of dopaminergic function. Indeed, reduction in sEBR following dopamine antagonist administration as well as an increase after dopamine agonist administration was observed [18,19,20,21]. sEBR was also linked to changes in dopamine regulation in several neurological and psychiatric disorders [22,23,24], including substance use disorders [25,26]. Previous studies have also shown a relationship between sEBR and cognitive functions such as attention and cognitive flexibility [27]. Moreover, a recent study by Mathar et al. [28] identified association of sEBR with gambling severity. Importantly, sEBR has shown effective reliability as a measure of trait-like differences in dopaminergic transmission [29]. The sEBR method is easily implemented and affordable, thus suitable for fast monitoring and execution of large-scale studies. The potential link between sEBR and dopaminergic activity makes it of particular interest for clinical use, including behavioral addictions.

In this pilot study, we used sEBR to examine the potential link between individual differences in dopaminergic neurotransmission and PIU patterns in healthy participants. To the best of our knowledge, sEBR has never been linked to problematic internet behavior before. However, based on the negative association of gambling severity and sEBR in gamblers, as reported by Mathar et al. [28], we hypothesized to observe a negative association between sEBR and PIU.

## 2. Materials and Methods

### 2.1. Subjects and Procedures

In sum, 62 participants (median age 25, IQR 6 years, 34 females) without a reported history of psychiatric disorders, neurological disorders, or psychotropic medication use were enrolled in the pilot study. This pilot study is part of a larger research project on problematic internet behavior. The call to participate was spread in the public media, university notice boards, and by word-of-mouth. Two groups of participants were created based on PIU symptom levels: a group with the most expressed PIU symptoms (PIU group), and a control group with the least prevailed problematic internet behavior. Demographic characteristics of the sample are presented in Table 1. Subjects were asked to abstain from alcohol for 24 h prior to the testing and did not consume nicotine and caffeine-containing drinks for at least two hours prior to the experiment. Subjects with corrected to normal vision were instructed to wear glasses rather than lenses. The study was approved by the Vilnius Regional Biomedical Research Ethics Committee (Nr.2019/10-1159-649), and all participants gave their written informed consent.

### 2.2. Psychological Assessment Included

Investigation of internet usage pattern was estimated based on the Problematic Internet Use Questionnaire, 9 items version (PIUQ-9) [30]. Subjects were also assessed for experienced levels of depression (BDI-II, Beck Depression Inventory, Second Edition [31]), anxiety (BAI, Beck Anxiety Inventory [32]), and obsessive–compulsive symptoms (CBOCI, Clark–Beck Obsessive–Compulsive Inventory [33]). The level of impulsivity was measured using the Barratt Impulsiveness Scale (BIS-11 [34]).

#### 2.2.1. Problematic Internet Use Questionnaire, 9 Items Version

The PIUQ-9 is a short self-report instrument designed to measure PIU severity. Nine items were evaluated using a five-point Likert scale, ranging from “Never” (1) to “Always/Almost always” (5) with higher scores indicating increased risk for problematic behavior. Based on the previous psychometric characteristics study in a sample of Lithuanian students [30], a cut-off value of >20 was used for screening markedly expressed PIU symptoms. Cronbach’s alpha of the scale in the current sample was 0.87.

#### 2.2.2. Barratt Impulsiveness Scale

The BIS-11 is a self-report scale that assesses personality and behavioral aspects of impulsivity [35]. It has 30 items that are scored on a four-point Likert type scale ranging from “Rarely/Never” (1) to “Almost always/Always” (4). Higher scores indicate higher levels of impulsivity. The Lithuanian version of the BIS-11 demonstrated good construct validity, appropriate internal consistency, test–retest reliability, and prognostic value of BIS-11 in predicting addictive and delinquent behaviors such as smoking, alcohol consumption, and law breaking [36]. Cronbach’s alpha of the scale in the current sample was 0.79.

#### 2.2.3. Beck Depression Inventory II

The BDI-II is a 21-item self-report measure designed to measure the severity of depression [31]. The BDI-II is regarded as a cost-effective tool to measure the severity of depression, which is widely applicable for both research and clinical settings worldwide [37,38,39]. Responses to each of the 21 items on the BDI-II range from 0 to 3. A total score is obtained, with higher scores suggesting a greater severity of depression. Cronbach’s alpha of the scale in the current sample was 0.87.

#### 2.2.4. Beck Anxiety Inventory

The BAI is a self-report measure designed to assess the intensity of physical and cognitive anxiety symptoms. The scale is composed of 21 items scored on a four-point Likert scale, ranging from “Not at all” (0) to “Severely” (3), with higher total score representing greater anxiety severity. Cronbach’s alpha of the scale in the current sample was 0.83.

#### 2.2.5. Clark–Beck Obsessive–Compulsive Inventory

A 25-item CBOCI was used to assess the severity of obsessive and compulsive symptoms based on the two respective subscales. In this questionnaire, scores are collected on a four-point Likert scale, with a higher score in each subscale indicating greater severity of obsessive and compulsive symptoms. Cronbach’s alpha of the scale in the current sample was 0.87.

### 2.3. sEBR Assessment

Participants sat in front of a computer screen and were instructed to move as little as possible. A fixation cross at approximately 0.8 m distance was presented while subjects were monitored during sEBR assessment to ensure that they were focusing on the cross shown on the computer screen as instructed. Data collection continued for 5 min [40,41].

Spontaneous eye blinking rates (sEBRs) were assessed with electrooculograms (VEOG and HEOG) recorded from above and below the left eye and from the right and left outer canthi. The concurrent EEG recording was performed with an ANT device (ANT Neuro, Hengelo, The Netherlands) and a 64 channel WaveGuard EEG cap (International 10–20 System) with Ag/AgCl electrodes, where mastoids served as a reference, and the ground electrode was attached close to Fz. Impedance was kept below 20 kΩ, and the sampling rate was set at 2048 Hz.

For sEBR extraction, we implemented an automated pipeline provided by MATLAB toolbox BLINKER [42]. The spontaneous blinking rate was defined as the number of blinks per minute and computed as the average blink rate for the 5 min dataset as the number of blink maxima divided by the total length of the dataset in minutes.

### 2.4. Statistical Evaluation

The data were not normally distributed; thus, nonparametric statistical tests were applied. Scores on BIS-11, BDI-II, BAI, CBOCI, and sEBR were compared between groups based on PIUQ-9 cut-off scores (>20) using the Independent Samples Mann–Whitney Test. Spearman’s correlation coefficients were calculated to assess the associations between PIUQ-9 scores, sEBR, and scores on BIS-11, BDI-II, BAI, and CBOCI in the whole sample, and separately in a group with PIU for in-depth exploration. Bonferroni correction for multiple comparisons was applied to reduce the likelihood of Type I error [43]. *p* Values less than 0.007 (0.05/7) were regarded as significant. Statistical evaluation was performed using SPSSv20 (SPSS Inc., Chicago, IL, USA).

## 3. Results

Based on PIUQ-9 scores, 32 subjects who scored on PIUQ-9 > 20 were assigned to the PIU group. A control group consisted of 30 subjects who scored on PIUQ-9 ≤ 16. Medians, interquartile ranges, and statistical comparison outcomes are presented in Table 1. When compared with controls, individuals with PIU differed significantly on BIS-11 and on the scores of CBOCI. No other statistically significant differences on scale scores were observed between the groups. In the combined sample, a positive correlation between PIUQ-9 scores and scores on BIS-11 and CBOCI was detected (Table 2). In addition, a marginal association to BDI-II and BAI scores was seen.

**Figure 1:**
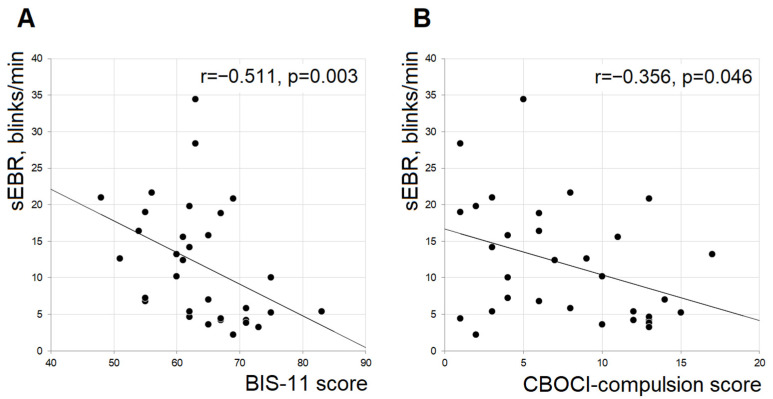
(**A**) Scatterplot of BIS-11 scores against individual sEBR values in PIU group; (**B**) scatterplot of CBOCI-compulsion scores against individual sEBR values in PIU group.

Average blinking rates over the collection ranged from 1.4 to 34.4 blinks/min, falling within the range of values reported in the literature [44,45,46] for spontaneous blinking rates. Spontaneous eye blinking rates did not differ between the groups: median C 10.1, IQR C 13.5; median PIU 7.8, IQR PIU 8.8; U = 520.50, *p* = 0.568. However, sEBR showed a sign of negative correlation to BIS-11 scores (r = −0.284, *p* = 0.025) in a combined sample, and this relationship was significant in a PIU group (r = −0.511, *p* = 0.003; Figure 1A). Moreover, in the PIU group, exploratory evaluation revealed a negative association between sEBR and compulsive symptoms (r = −0.356, *p* = 0.046; Figure 1B) that did not survive correction for multiple comparisons. No other associations were observed.

## 4. Discussion

With increasing amounts of problematic internet behavior, research is focused on the neural indices that can be used for diagnostic and monitoring purposes. Nevertheless, for a potential marker to be suitable, it has to be sensitive to subtle changes in brain activity. Additionally, the method that is easily implemented and cheap has larger potential to be widely applied. Modern neuroimaging methods, though enabling monitoring of various brain functioning aspects, are mostly expensive and require highly demanding qualifications. Spontaneous eye blinking rate, in comparison, is regarded as a non-invasive, cheap method indexing dopaminergic function. Since internet addiction was previously related to substance use disorders and associated with changes in dopaminergic transmission, we hypothesized that spontaneous eye blinking rate—a non-invasive predictor of dopamine functioning—might be a sensitive marker that would be capable of distinguishing problematic use from healthy/functional internet use.

Many studies enroll subjects with diagnosed mental health issues [47,48,49]. We, however, focused on the non-clinical sample of adults and assessed excessive internet use patterns alongside self-reported depression, anxiety, and impulsivity levels, as well as the frequency and severity of obsessive and compulsive symptoms. As expected, the group with markedly expressed PIU symptoms experienced higher levels of impulsivity and compulsive symptoms but did not differ in depression and anxiety levels from the control group. This result suggests that both groups experienced similar mental distress levels. It has been shown that mental distress (i.e., anxiety and depressive symptoms) might be a possible perpetuating factor in predicting increased levels of problematic internet behavior [50,51]. However, in our recent study [52], we showed that relationships between PIU, anxiety, and depressive symptoms are mediated via impulsivity. In the current study, it appears that increased impulsivity levels distinguish the PIU group from controls more easily than depressive or anxiety symptoms, and this is also reflected in the positive correlation between PIUQ-9 and BIS-11 scores. Importantly, we observed elevated compulsivity in the PIU group as compared to controls, and that the combined sample PIUQ-9 scores positively correlated to CBOCI scores. This was the expected observation as several studies found that obsessive-compulsive symptoms are the most related symptoms in internet addicts [53,54,55].

Contrary to our expectations, the two groups did not differ in blinking rates. There is limited knowledge on the sEBR changes in behavioral addictions. However, similar blinking rates in the PIU group and controls are in line with a recent observation by Mathar et al. [28] in problematic gamblers and controls. The authors observed that the gambling severity correlated negatively, and the overall psychopathology correlated positively with sEBR in gamblers, but not in healthy controls. We could not observe any relationship between internet use patterns and sEBR in the PIU group. It might be that the PIU group was not that significantly affected to show pronounced disturbance in dopaminergic transmission. Indeed, subjects enrolled in the current study were of generally good health, mostly experiencing minimal to moderate levels of depression, and anxiety (Table 1). Self-reported perceived impairment related to internet usage is a strong predictor of general PIU psychopathology [56,57]. However, problematic use (as assessed in the current study) and true clinical impairment due to problematic behavior, while overlapping, are still two different concepts and potentially should cause different levels of functional alterations. Previously shown changes of sEBR in addicted subjects [25,26] might be due to a relatively stronger impairment in dopamine functioning. Importantly, excessive internet use also incorporates a range of online activities including gambling, gaming, streaming, pornography, impulsive buying, and social networking, which if used excessively might be considered problematic [58]. Our sample size precluded us from the analysis in different subgroups of PIU. Thus, further studies should address this issue focusing on particular groups with specific problematic aspects in one area.

A significant negative correlation between blinking rates and BIS-11 scores was observed in the PIU group along with a similar trend in the combined sample. The observation of negative association between impulsivity and sEBR was in line with Korponay et al. [46] and findings from pharmacological studies [59]. Moreover, although not surviving comparison for multiple testing, the blinking rate in the PIU group was negatively associated with the scores on the compulsive subscale of CBOCI, indicating that subjects experiencing more compulsive behaviors might have lower sEBR. Compulsions resemble a form of inflexible behavior [60,61,62]. A higher blinking rate was previously associated with enhanced cognitive flexibility [63,64], contributing to reinforcement of certain behaviors depending on the environmental demands [45]. Van Slooten et al. [45] suggested that higher sEBR, as a potential indicator of higher tonic dopamine levels, may reflect increased energetic demands for promotion of the exploratory behaviors towards novel options; lower sEBR, in turn, potentially reflecting lower tonic dopamine levels, may relate to energy conservation for a selection of options with known reward. In line with that, a recent study by Seiler et al. [65] reported large individual differences in the compulsivity of mice associated with different strategies used by individual animals dealing with uncertainty on the experimental paradigm designed to track the development of habits and compulsions. Importantly, the authors showed that striatal dopamine signaling was a key part of the circuitry that drives compulsion [65]. Taking the abovementioned into account, a negative association between sEBR and compulsive symptoms in the PIU group might reflect on the subtle dopamine functioning alterations. However, it should be noted that recent work by Sescouse et al. [66] could not confirm the positive association between sEBR and dopamine synthesis capacity. Earlier, Dang et al. [67] were unable to show correlation with the dopamine D2 receptor availability. Thus, it is not fully clear which specific aspect of dopaminergic transmission (and to what extent) is indexed by sEBR. Although our observations precludes any strong conclusions about the biological mechanisms affecting spontaneous blinking rate in problematic internet behavior without any direct manipulations of the dopaminergic system, the initial result on relation of sEBR to compulsive symptoms in the high internet use group is promising and should be investigated further in larger samples.

## 5. Conclusions

To summarize, we did not find a difference in spontaneous eye blinking rates between the group with problematic internet use and the control group. Nevertheless, the group with the problematic internet behavior expressed higher levels of impulsivity and compulsive behavior symptoms but did not differ in depression and anxiety levels from controls, indicating similar levels of mental distress between groups. In the PIU group, impulsivity levels were inversely related to sEBR, and a sign of negative association to compulsive behavior was observed. Future research should enroll subjects with high PIU and strongly expressed psychopathology levels to further address the utility of sEBR as a potential biomarker.

## Figures and Tables

**Table 1:** Demographic and psychological characteristics of the study groups.

	PIU ^1^	Control	Mann–Whitney U	*p*
F/M	20/12	14/16		
Age	25 (7.0)	24 (5.0)	544.00	0.365
PIUQ-9	24 (5.0)	14 (3.0)	740.00	<0.001
BIS-11	63 (9.0)	57 (7.5)	700.00	0.002
BDI-II	8 (9.8)	4 (9.0)	594.00	0.108
BAI	29 (8.8)	26 (6.0)	568.50	0.130
CBOCI	22.5 (13.8)	12.0 (7.0)	740.00	<0.001

^1^ As defined by 9 items version Problematic Internet Use Questionnaire, cut-off score > 20; BIS-11—Barratt Impulsiveness Scale; BDI-II—Beck Depression Inventory, Second Edition; BAI—Beck Anxiety Inventory; CBOCI, Clark–Beck Obsessive–Compulsive Inventory. Medians and interquartile ranges are reported.

**Table 2:** Association of the PIU level and sEBR with selected tests of psychological assessment.

		sEBR	PIUQ-9	BIS-11	BDI-II	BAI	CBOCI
PIUQ-9	r	0.009	1.000	0.361	0.252	0.240	0.437
	*p*	0.942		0.004	0.048	0.062	<0.001
sEBR	r	1.000	0.009	−0.284	−0.155	−0.040	−0.038
	*p*		0.942	0.025	0.228	0.758	0.768

PIUQ-9—Problematic Internet Use Questionnaire, 9 items version; sEBR—spontaneous eye blinking rate; BIS-11—Barratt Impulsiveness Scale; BDI-II—Beck Depression Inventory, Second Edition; BAI—Beck Anxiety Inventory; CBOCI, Clark–Beck Obsessive–Compulsive Inventory.

## Data Availability

The data presented in this study are available on request from the corresponding author. The data are not publicly available due to privacy restrictions.
